# Immunoprofiling at an Institutional Scale Reveals That High Numbers of Intratumoral CD8^+^ and PD-1^+^ Cells Predict Superior Patient Survival Across Major Cancer Types Independent of Major Risk Factors

**DOI:** 10.1200/PO-25-00240

**Published:** 2025-09-04

**Authors:** Joao V. Alessi, James R. Lindsay, Anita Giobbie-Hurder, Bijaya Sharma, Kristen Felt, Priti Kumari, Tali Mazor, Ethan Cerami, William Lotter, Jennifer Altreuter, Jason Weirather, Ian Dryg, Katharina Hoebel, Michael Manos, Elio Adib, Jennifer D. Curtis, Biagio Ricciuti, Alessandro Di Federico, Fatme Ghandour, Eddy Saad, Xin-an Wang, Federica Pecci, Marta Holovatska, Malini M. Gandhi, Melissa E. Hughes, Tess A. O'Meara, Sabrina J. Chan, Kathleen Pfaff, Panagiotis A. Konstantinopoulos, F. Stephan Hodi, Margaret A. Shipp, Sabina Signoretti, Toni Choueiri, Xiao X. Wei, Sandro Santagata, Glenn J. Hanna, Nancy U. Lin, Sara M. Tolaney, Joyce Liu, Peter K. Sorger, Neal Lindeman, Lynette M. Sholl, Jonathan A. Nowak, David Barbie, Mark M. Awad, Bruce E. Johnson, Scott J. Rodig

**Affiliations:** ^1^ Department of Medical Oncology, Dana-Farber Cancer Institute, Boston, MA; ^2^ Department of Data Science, Dana-Farber Cancer Institute, Boston, MA; ^3^ Center for Immuno-Oncology, Dana-Farber Cancer Institute, Boston, MA; ^4^ Department of Pathology, Brigham & Women's Hospital, Boston, MA; ^5^ Department of Biostatistics and Environmental Health, Harvard T.H. Chan School of Public Health, Boston, MA; ^6^ Laboratory for Systems Pharmacology, Harvard Medical School, Boston, MA

## Abstract

**PURPOSE:**

Retrospective studies have found associations between the number of intratumoral immune cells and patient outcomes for specific cancers treated with targeted therapies. However, the clinical value of routinely quantifying intratumoral immune biomarkers using a digital pathology platform in the pan-cancer setting within an active clinical laboratory has not been established.

**METHODS:**

We developed ImmunoProfile, a daily clinical workflow that integrates automated multiplex immunofluorescence tissue staining, digital slide imaging, and machine learning–assisted scoring to quantify intratumoral CD8^+^, PD-1^+^, CD8^+^PD-1^+^, and FOXP3^+^ immune cells and PD-L1 expression in formalin-fixed, paraffin-embedded tissue samples in a standardized and reproducible manner. We prospectively applied ImmunoProfile to biopsies collected from 2,023 unselected patients with cancer over a 3-year period in the clinical laboratory and correlated the results with patient survival.

**RESULTS:**

In the pan-cancer cohort, patients with high numbers of intratumoral CD8^+^ or PD-1^+^ cells in had significantly lower risks of death compared with those with low numbers (CD8^+^: high *v* low hazard ratio [HR], 0.62 [95% CI, 0.48 to 0.81], Wald *P* = .002; PD-1^+^: high *v* low HR, 0.65 [95% CI, 0.51 to 0.83]; *P* = .0009) after adjusting for risk factors, including cancer type. In subset analyses, patients with high numbers of intratumoral CD8^+^, PD-1^+^, and/or CD8^+^PD-1^+^ cells showed lower risks of death from non–small cell lung, colorectal, breast, esophagogastric, head and neck, pancreatic, and ovarian cancers after considering clinical risk factors, including American Joint Committee on Cancer stage, and despite varying therapies (all *P* < .05).

**CONCLUSION:**

Routinely quantifying intratumoral CD8^+^ and PD-1^+^ cells with a clinically validated digital pathology platform predicts patient survival across major cancer types, independent of clinical stage and despite diverse treatment regimens.

## INTRODUCTION

The foundation for cancer diagnoses remains the examination of tissue histomorphology using hematoxylin and eosin (H&E)–stained formalin-fixed paraffin-embedded (FFPE) tissue sections. These observations are supported by immunohistochemical staining (IHC) results and genetic testing to finalize tumor subclassification, staging, and direct therapy.^1-4^

**CONTEXT**

**Key Objective**
We aimed to (1) evaluate the feasibility of implementing a new digital pathology workflow that quantifies intratumoral immune biomarkers into daily clinical practice, and (2) determine whether quantified intratumoral immune biomarkers, obtained in real time, provide prognostic survival information for patients with cancer.
**Knowledge Generated**
Immunoprofiling conducted on over 2,000 patient samples over 3 years was successful more than 96% of the time. Patients with high levels of intratumoral CD8^+^, PD-1^+^, and/or CD8^+^PD-1^+^ cells exhibited lower risks of death from non–small cell lung, colorectal, breast, esophagogastric, head and neck, pancreatic, and ovarian cancers, independent of clinical risk factors, including American Joint Committee on Cancer stage, and despite various therapies.
**Relevance**
The routine use of a digital pathology platform to quantify intratumoral immune cell populations yields prognostic survival information that is not currently incorporated into clinical practice.

The tumor microenvironment (TME) sampled in FFPE tissue sections contains architectural, cellular, and molecular features that remain unreported despite their potential clinical importance.^5^ Of these, immune biomarkers are of greatest interest.^6,7^ Retrospective research studies have shown that the density of tumor-infiltrating lymphocytes is correlated with outcomes within and across cancer types.^8-10^ These include the numbers of intratumoral CD8^+^ and PD-1^+^ T cells, which indicate an active immune response, and FOXP3^+^ T cells, which promote immune suppression.^7^ Nevertheless, scoring immunologic features has not been integrated into routine clinical practice, primarily because of the time and effort needed to quantify immune cell populations.^11,12^

One exception is scoring PD-L1, a PD-1 ligand that enforces T-cell exhaustion.^13^ PD-L1 expression in tissue samples is linked to a favorable response to PD-1/PD-L1 inhibitors, and PD-L1 IHC is routinely conducted.^14^ However, pathologists score PD-L1 IHC subjectively, so its assessment, accuracy, and reproducibility have been questioned.^15,16^

Automated multiplexed immunostaining, digital imaging, and image analysis platforms have the potential to facilitate and standardize the reporting of critical immune biomarkers in tissue samples. However, before such tests can be implemented in clinical practice, they must comply with strict clinical laboratory standards and undergo clinical validation, ideally through prospective trials.^17-20^ Only Immunoscore, a semiautomated platform that quantifies CD3^+^ and CD8^+^ T cells in tissue sections, has met these requirements for immune markers.^21-24^ Immunoscore provides prognostic information that traditional diagnostic and staging criteria do not capture for patients with colorectal carcinoma (CRC), serving as a complement to the conventional TNM staging system. Immunoscore has not been applied to cancers beyond CRC.

We leveraged advances in automated multiplex immunofluorescence (mIF) staining,^25-27^ digital whole-slide image (WSI) capture,^28-30^ and machine learning (ML)–based automated scoring^31,32^ to develop and validate a new workflow, termed ImmunoProfile, for the clinical laboratory. We used ImmunoProfile to prospectively capture and quantify four key biomarkers associated with an active (CD8 and PD-1) and suppressed (FOXP3 and PD-L1) immune response in FFPE tissues from 2,023 patients presenting to a tertiary care cancer center over 3.25 years. We find that we can quantitatively and reproducibly capture and report these key components of the TME in a standardized format. The intratumoral counts of CD8^+^, PD-1^+^, and/or CD8^+^PD-1^+^ cells predicted overall survival (OS) for patients across major cancer types, independent of clinical stage and despite diverse therapies.

## METHODS

### Clinical Workflow and Validation

**FIG 1.**
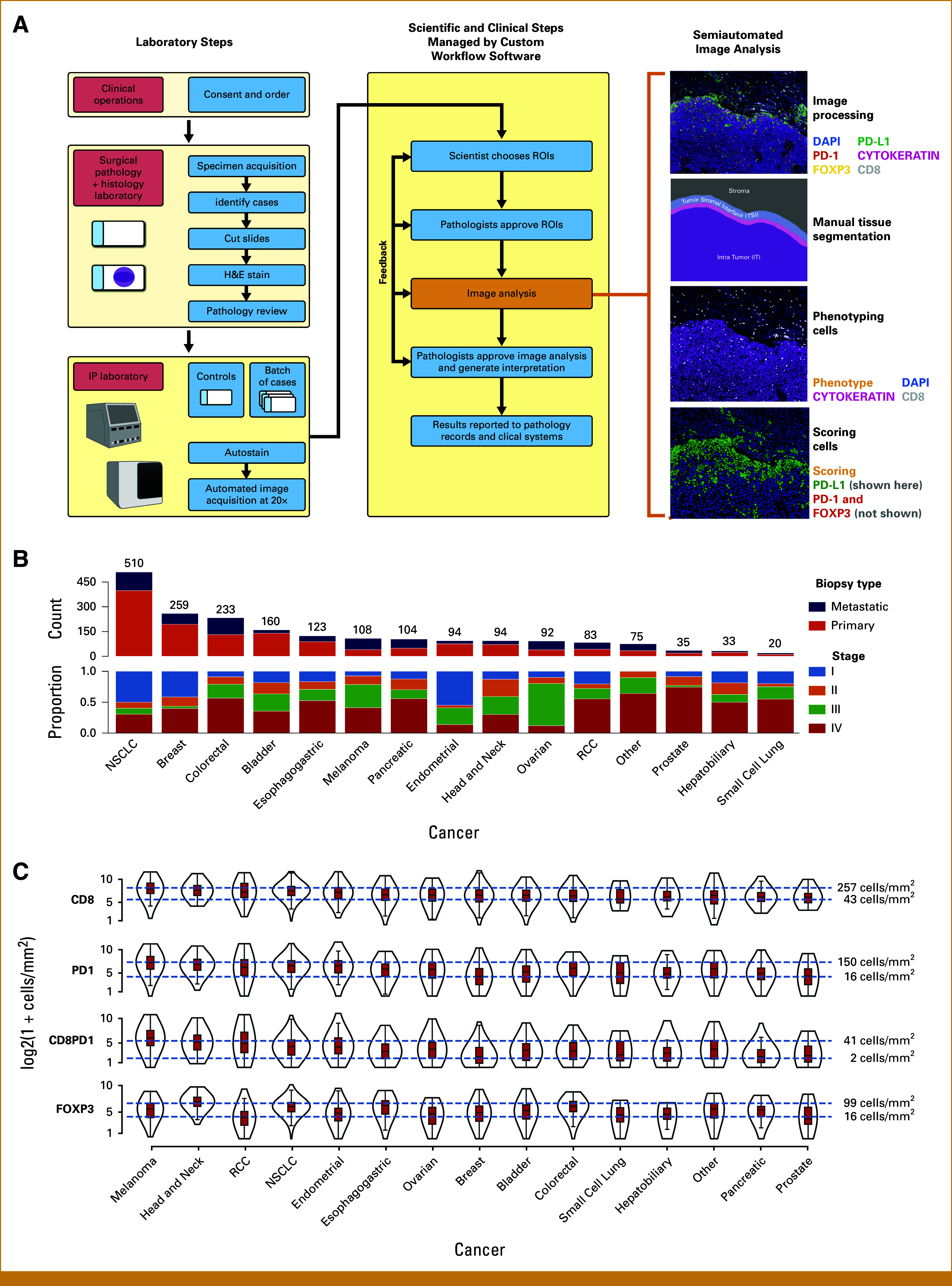
(A) Schematic outlining the ImmunoProfile workflow in the CLIA-certified laboratory. Consented patients and their diagnostic pathology material were first identified. Slides were cut to generate an H&E-stained slide, which a pathologist reviewed and confirmed to contain diagnostic material. An additional slide was stained on an autostainer and scanned, and a multiplex digital image of the stained tissue was generated. Technicians digitally annotated the images, including selecting ROIs and demarcating the tumor border under pathologists' review and approval. This was followed by algorithmic training to identify positive and negative staining cells for each biomarker and automated quantitative scoring using image analysis software. A pathologist approved and signed out the image analysis results and the final scores. Results were reported to a clinically accessible database. Images and image analysis results were retained in a HIPAA-compliant data warehouse. Further details are provided in Supplementary Methods. (B) The 2,023-patient pan-cancer ImmunoProfile cohort. The cases are separated into 14 major diagnostic categories and other rare cancer types. The number of cases within each major cancer type is further annotated by the tissue acquisition site (primary *v* metastatic) and patients' clinical stage. Further details are provided in the Data Supplement. (C) Violin plots showing the distributions of intratumoral immune cell densities (log_2_ of cells per mm^2^) for all cases, separated according to cancer type and organized from the highest to lowest average CD8^+^ immune cells. The blue lines indicate the immune cell densities at the 75th and 25th percentiles for the pan-cancer cohort. The black lines represent the mean value, the red boxes represent the 75th and 25th percentile thresholds, and the black outlines represent case numbers at the indicated density for each cancer type. Note that there are cases within the highest and lowest quartiles for each biomarker for each cancer type. CLIA, clinical laboratory improvement amendments; H&E, hematoxylin and eosin; NSCLC, non–small cell lung carcinoma; RCC, renal cell carcinoma; ROIs, regions of interest.

ImmunoProfile was validated within a clinical laboratory improvement amendments (CLIA)-certified pathology laboratory (Fig 1A, Data Supplement, Figs S1 and S2, Supplementary Methods and Materials). The assay was performed prospectively on all cases, upon request by clinical oncologists, on all consented patients (DFCI protocols 11-104, 17-000; 22-176), using available clinical materials. An H&E slide from the best tumor block was reviewed to ensure a minimum of 100 tumor cells. An additional slide was used for automated mIF staining with a Leica BOND Rx and imaged on an Akoya Polaris at 200×.^30,33,34^ At least three and at most six regions of interest (ROIs) were selected per WSI using Inform software on the basis of predetermined metrics. Technicians trained a random forest–based ML algorithm in Inform using representative cells and used the algorithm to quantify defined cell types. Custom tools^35^ were used to calculate cell densities,^36,37^ and PD-L1 tumor proportion score (TPS) and combined proportion score^36,37^ (CPS, Data Supplement). An expert pathologist signed out each case.

### Statistical Methods and Prognostic Models

Patient information was obtained from medical records and the DFCI OncDRS data warehouse with IRB approval (Data Supplement, Table S1).^38^ Appropriate clinical experts manually reviewed every case.

For an unbiased approach, we divided patients within each analyzed group by tertiles, categorizing them as high, intermediate, and low for each biomarker when there were more than 100 patients and over 50 deaths. We divided patients at the median into high and low categories for each biomarker when the group did not fulfill the above criteria. Threshold values are listed in the Data Supplement (Table S2).

Comparisons for continuous scaled data used Wilcoxon rank-sum or Kruskal-Wallis tests. Categorical comparisons used Fisher's exact or chi-squared tests. Statistical significance was defined as *P* ≤ .05. OS was from the date of tissue acquisition to the date of death from any cause. The patients alive at the time of data retrieval were censored at the last date of contact. OS was estimated using the Kaplan-Meier method and compared with log-rank testing; 95% CIs were calculated using the log(-log) methodology.

Associations between biomarker measurements and OS were assessed using Cox proportional-hazards models adjusted for clinical risk factors selected before analyses.^39^ To avoid overfitting, the number of risk factors included in each model was adjusted according to the cohort size and the number of deaths. Immune cells with adjusted Wald *P* values ≤.1 from the single-marker analyses were considered candidates in the multivariable setting (Supplementary Methods).

The IRB of the Dana-Farber Cancer Institute provided ethical approval for this work.

## RESULTS

### ImmunoProfile Reliably Quantifies Key Immunologic Biomarkers in Clinical Samples

We modified a research protocol^36^ to develop a new clinical workflow, ImmunoProfile, which integrates automated mIF tissue staining, digital WSI capture, and ML-directed analysis to calculate CD8^+^, PD-1^+^, CD8^+^PD-1^+^, FOXP3^+^ cell densities, and PD-L1 TPS and CPS in FFPE tissue sections within a CLIA-certified laboratory (Fig 1A). Validation studies showed a high concordance between ImmunoProfile and IHC (Data Supplement, Fig S1). The application of ImmunoProfile to clinical samples revealed minimal variability in staining, imaging, and scoring over time (Data Supplement, Fig S2). Fewer than 3% of cases failed for technical reasons (Supplementary Methods). The entire multistep workflow met or exceeded the laboratory's quality-control requirements and received approval for clinical application.

### High Intratumoral Immune Cell Counts and PD-L1 Expression Are Found Across Cancer Types

In a prospective study, we analyzed all biopsy and resection tissue specimens sent for pathologic review from all consenting patients over 3.25 years. The final cohort of 2,023 cases included 14 major cancer types, with biopsies from both primary and metastatic sites, and from patients with low-stage disease and high-stage disease, as expected for a tertiary cancer center (Fig 1B).

We found that the numbers of intratumoral immune cells and PD-L1 expression varied significantly across cases and cancer types (Fig 1C). When we divided the cases on the basis of their biomarker scores into highest, middle, and lowest tertiles, we observed cases with scores in the top and bottom tertiles for each biomarker across cancer types (Data Supplement, Fig S3).

### Immune Biomarker Scores, Stratified by Tertiles, Predict Patient Survival in Pan-Cancer Analyses

At the study's completion, we examined the prognostic significance of the immune biomarker scores. Given the amount of data generated (more than 47 million cells analyzed across over 10,000 images), we present the most significant associations below. Additional data are listed in the Data Supplement.

**FIG 2.**
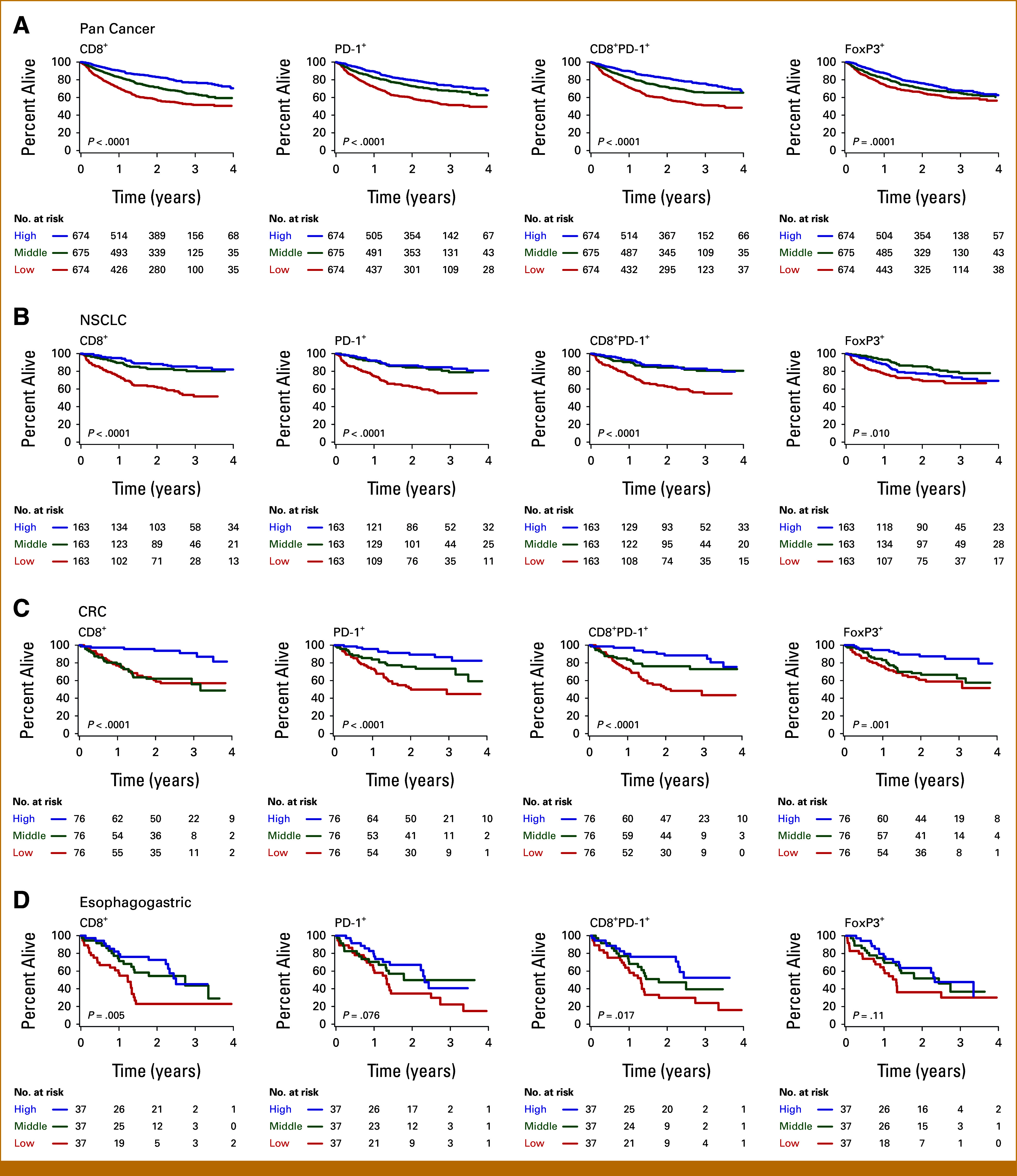
Kaplan-Meier estimates of OS with biomarker values divided into tertiles. The pan-cancer and major cancer subtypes were divided into high (blue), middle (green), and low (red) tertiles on the basis of their CD8^+^, PD-1^+^, CD8^+^PD-1^+^, and FoxP3^+^ immune cell densities, respectively, for each respective group. The number of patients at risk of death at the indicated time from diagnosis is indicated for (A) all patients (N = 2,023), and patients with (B) NSCLC (n = 489), (C) CRC (n = 228), and (D) esophagogastric carcinoma (EGD, n = 111). The *P* values by the log-rank test are indicated. CRC, colorectal carcinoma; NSCLC, non–small cell lung carcinoma; OS, overall survival.

In a pan-cancer analysis (N = 2,023; 632 deaths), we observed that patients had longer OS with high intratumoral CD8^+^, PD-1^+^, CD8^+^PD-1^+^, or FOXP3^+^ cell counts compared with those with intermediate or low cell counts (all log-rank *P* < .0001, Fig 2A). Patients also had better OS with high rather than low PD-L1 CPS (*P* = .002, Data Supplement, Fig S4A). Patients did not show significant survival differences when stratified by PD-L1 TPS (Data Supplement, Fig S4B).

### Immune Biomarker Scores Divided by Tertiles Predict OS for Major Cancer Types

For cancer type–specific cohorts encompassing more than 100 patients and 50 deaths, we divided patients into high, middle, and low groups on the basis of tertile splits in their respective biomarker score distributions (thresholds provided in the Data Supplement, Table S2). We found that patients with non–small cell lung carcinoma (NSCLC, n = 489), CRC (n = 228), and esophagogastric carcinoma (EGC, n = 111) had better OS with high compared with low intratumoral CD8^+^, PD-1^+^, and/or CD8^+^PD-1^+^ cells (log-rank *P* ≤ .05, Figs 2B-2D). Patients with NSCLC receiving immune checkpoint blockade (ICB, n = 114) or not receiving ICB (no-ICB, n = 372) had better OS with high CD8^+^, PD-1^+^, or CD8^+^PD-1^+^ cells (all *P* < .001, Data Supplement, Fig S5).

In additional analyses, patients with pancreatic carcinoma (PAN, n = 104) had better OS with high intratumoral PD-1^+^ cell counts compared with those with intermediate cell counts but not low cell counts (*P* = .03, Data Supplement, Fig S6A). This unusual result may reflect the distinct immune response profile associated with the cancer type.^40^ Patients with bladder carcinoma (BLAD, n = 159) did not show survival differences when split in this manner (Data Supplement, Fig S6B). None of these groups showed significant survival differences when stratified by PD-L1 CPS and TPS.

### Immune Biomarker Scores Divided at the Median Predict Overall Survival for Major Cancer Types

**FIG 3.**
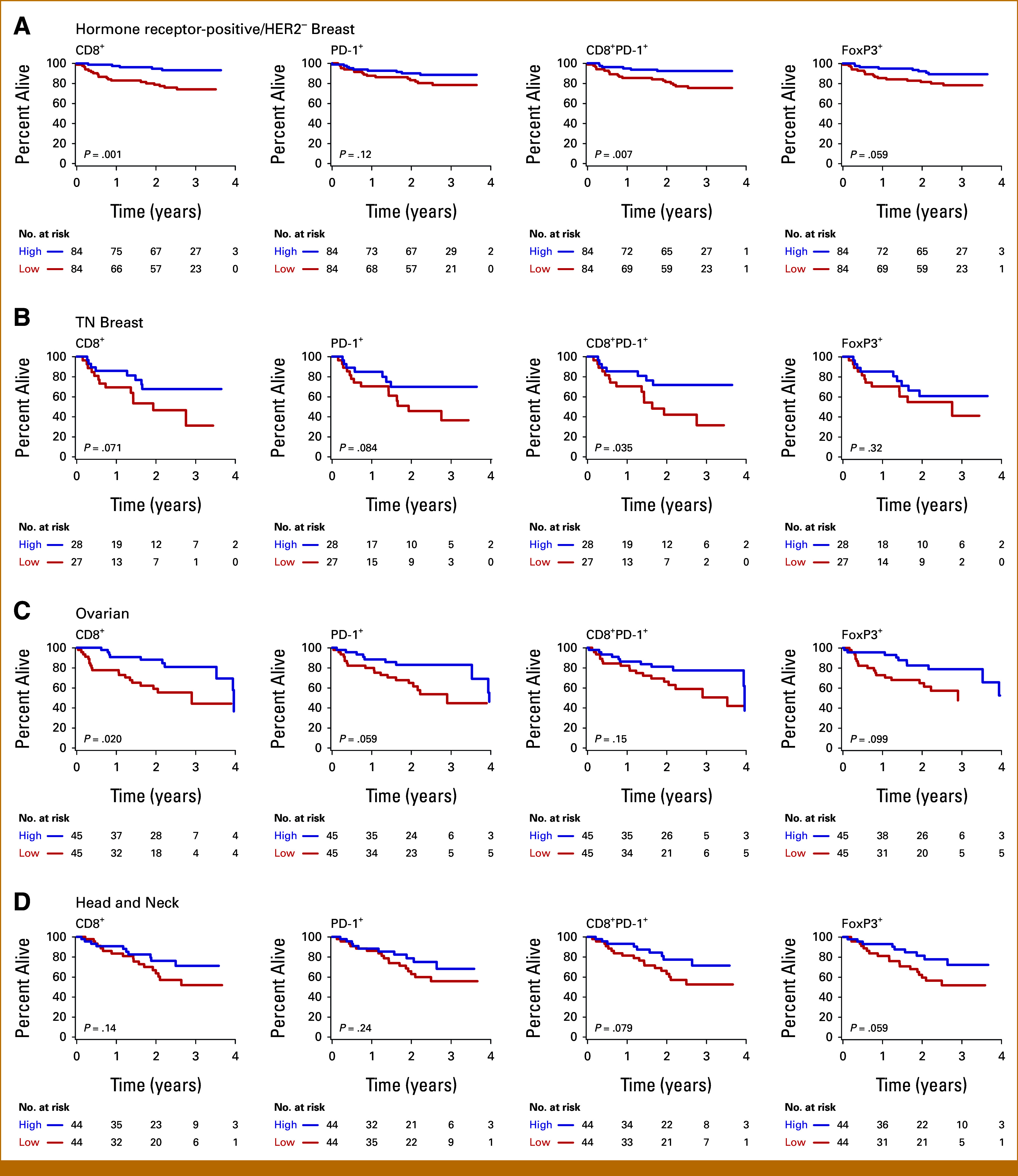
Kaplan-Meier estimates of OS for biomarker values divided at the median. The major cancer subtypes were divided according to their CD8^+^, PD-1^+^, CD8^+^PD-1^+^, and FoxP3^+^ immune cell densities into high (blue) and low (red) groups relative to each cancer type's median values. The number of patients at risk of death at the indicated time from diagnosis is indicated for patients with (A) hormone receptor–positive/HER2– breast carcinoma (n = 168), (B) TN breast carcinoma (n = 55), (C) ovarian carcinoma (n = 90), and (D) head and neck squamous cell carcinoma (n = 88). The *P* values by the log-rank test are indicated. HER2– Breast, human epidermal growth factor receptor 2–negative breast carcinoma; OS, overall survival; TN Breast, triple-negative breast carcinoma.

For cancer cohorts with <100 patients and/or 50 deaths, we divided patients into high and low groups on the basis of a split at the median in their respective biomarker score distributions. Patients with hormone receptor–positive/human epidermal growth factor receptor 2 (HER2)/neu–negative breast carcinoma (hormone receptor–positive/HER2– BRCA, n = 168), triple-negative breast carcinoma (TN BRCA, n = 55), and ovarian carcinoma (OVCA, n = 90) had significantly better OS with high compared with low CD8^+^ and/or CD8^+^PD-1^+^ cells (*P* < .05, Figs 3A-3C). Patients with head and neck squamous cell carcinoma (HNSCC, n = 88) trended toward better survival with high intratumoral CD8^+^PD-1^+^ cells (*P* = .08, Fig 3D). Patients with renal cell carcinoma (RCC, n = 79), endometrial carcinoma (ENDOM, n = 89), and cutaneous melanoma (MEL, n = 98) did not show significant survival differences by this analysis.

We next divided patients according to their PD-L1 scores. Patients with hormone receptor–positive/HER2– BRCA and high CPS had better OS compared with those with low CPS (*P* = .002; Data Supplement, Fig S7A). By contrast, patients with RCC and *high* TPS had *worse* OS than those with low TPS, as seen in retrospective studies^41^ (*P* = .049; Data Supplement, Fig S7B).

### Immune Biomarkers Predict Survival After Adjustments for Stage and Other Risk Factors

We next examined whether individual biomarkers predicted OS in a Cox model accounting for major clinical risk factors associated with survival, including cancer type, biopsy site, American Joint Committee on Cancer stage, age, sex, smoking status, and alcohol use (Data Supplement, Fig S8). Importantly, we selected the risk factors before calculating the results to avoid overfitting.

In the pan-cancer analysis, patients had better OS with high CD8^+^, PD-1^+^, CD8^+^PD-1^+^, or FOXP3^+^ cells after risk factor adjustments (HR range, 0.48-0.74, 95% CI range, 0.39 to 0.90; all *P* = .0001; Table 1; HRs listed in the Data Supplement, Table S3). In subgroup analyses, patients with NSCLC (with or without ICB), CRC, EGC, BLAD, hormone receptor–positive/HER2+ BRCA, HNSCC, and OVCA had better OS with high CD8^+^, PD-1^+^, CD8^+^PD-1^+^, and/or FOXP3^+^ cells, after risk factor adjustments (Table 1; Data Supplement, Table S3).

**TABLE 1.** Biomarker Associations With Overall Survival From Univariate Cox Models With Clinical Risk Factor Adjustments

Cancer Type	Patients/Deaths	Division	*P*				Risk Factor Adjustments
			CD8^+^	PD-1^+^	CD8+PD-1^+^	FOXP3^+^	
Pan-cancer	2,023/632	Tertile	**.0001**	**.0001**	**.0001**	**.0001**	Cancer type, primary/met, smoking, alcohol, age, sex
NSCLC (all)	489/115	Tertile	**.009**	**.001**	**.0001**	.31	AJCC stage, smoking, alcohol, age, sex
NSCLC (ICB)	117/54	Tertile	**.001**	**.0002**	**.04**	**.03**	AJCC stage, smoking, alcohol, age, sex
NSCLC (No-ICB)	372/61	Tertile	.07	.08	**.003**	.19	AJCC stage, smoking, alcohol, age, sex
CRC	228/66	Tertile	**.006**	**.0006**	**.006**	.27	AJCC stage, MSI status, smoking, alcohol, age
PANC	104/71	Tertile	.23	**.007**	.48	**.04**	AJCC stage, smoking, alcohol, age
EGC	111/56	Tertile	**.02**	.12	.08	.36	AJCC stage, smoking, alcohol, age
BLAD	159/57	Tertile	.69	.80	.75	**.03**	AJCC stage, smoking, alcohol, age, sex
Hormone receptor–positive/HER2– BRCA	168/25	Median	**.002**	.24	**.02**	**.05**	Low (I/II) or high (III/IV) stage
TN BRCA	55/21	Median	.35	.24	.06	.78	Low (I/II) or high (III/IV) stage
HNSCC	88/27	Median	.14	.27	**.05**	.26	Low (I/II) or high (III/IV) stage
OVCA	90/30	Median	**.02**	.06	.09	**.05**	Primary *v* metastatic disease
RCC	79/24	Median	.97	.96	.25	.90	AJCC stage
ENDOM	89/19	Median	.35	.30	.18	.43	Primary *v* metastatic disease
Cut. MEL	98/18	Median	.52	.45	.54	.83	AJCC stage

NOTE. The hazard ratio and 95% CI values for all comparisons with *P* values ≤.05 are presented in the Data Supplement (Table S3). Bold indicates *P* ≤ .05.

Abbreviations: AJCC, American Joint Committee on Cancer; BLAD, bladder carcinoma; CRC, colorectal carcinoma; cut. MEL, cutaneous melanoma; EGC, esophagogastric carcinoma; ENDOM, endometrial carcinoma; HER2– BRCA, HER2-negative breast carcinoma; HNSCC, head and neck squamous cell carcinoma; ICB, treatment with immune checkpoint blockade; MSI, microsatellite instability.; no-ICB, no treatment with checkpoint blockade; NSCLC, non–small cell lung cancer; OVCA, ovarian carcinoma; PANC, pancreatic carcinoma; RCC, renal cell carcinoma; TN BRCA, triple-negative breast carcinoma.

For patients with pancreatic carcinoma (PANC), there was better OS with either high or low numbers of intratumoral PD-1+ cells, but not intermediate numbers (*P* = .007). Patients with TN BRCA trended toward better OS with high CD8^+^PD-1^+^ cells (*P* = .06, Table 1). Patients with RCC, ENDOM, and cutaneous MEL did not show significant differences in OS in this analysis incorporating risk adjustments (Table 1).

In the pan-cancer analysis, no significant differences in OS were observed according to PD-L1 scores. However, patients with NSCLC treated with ICB had superior OS with high PD-L1 CPS or TPS (>1%; *P* = .04, .05; Data Supplement, Table S4). Similarly, patients with CRC had superior OS with high TPS (>5%) (*P* = .02, Data Supplement, Table S4). By contrast, patients with RCC had *worse* OS with *high* TPS, after risk factor adjustments (*P* = .04, Data Supplement, Table S4).

### Multivariable Biomarker Analysis Identifies Specific Immune Cell Types That Are Independently Associated With OS

Numbers of CD8^+^, PD-1^+^, and CD8^+^PD-1^+^ cells were correlated across cases (r = 0.8-0.92, Data Supplement, Fig S9). The numbers of FOXP3^+^ cells and PD-L1 scores were less correlated (r = 0.26-0.63). Therefore, we investigated which individual immune biomarker(s) were independently associated with the risk of death using multivariable Cox biomarker modeling. Briefly, we began by using the biomarker that best predicted survival and then added additional biomarkers in an attempt to improve predictive power (see Methods).

**FIG 4.**
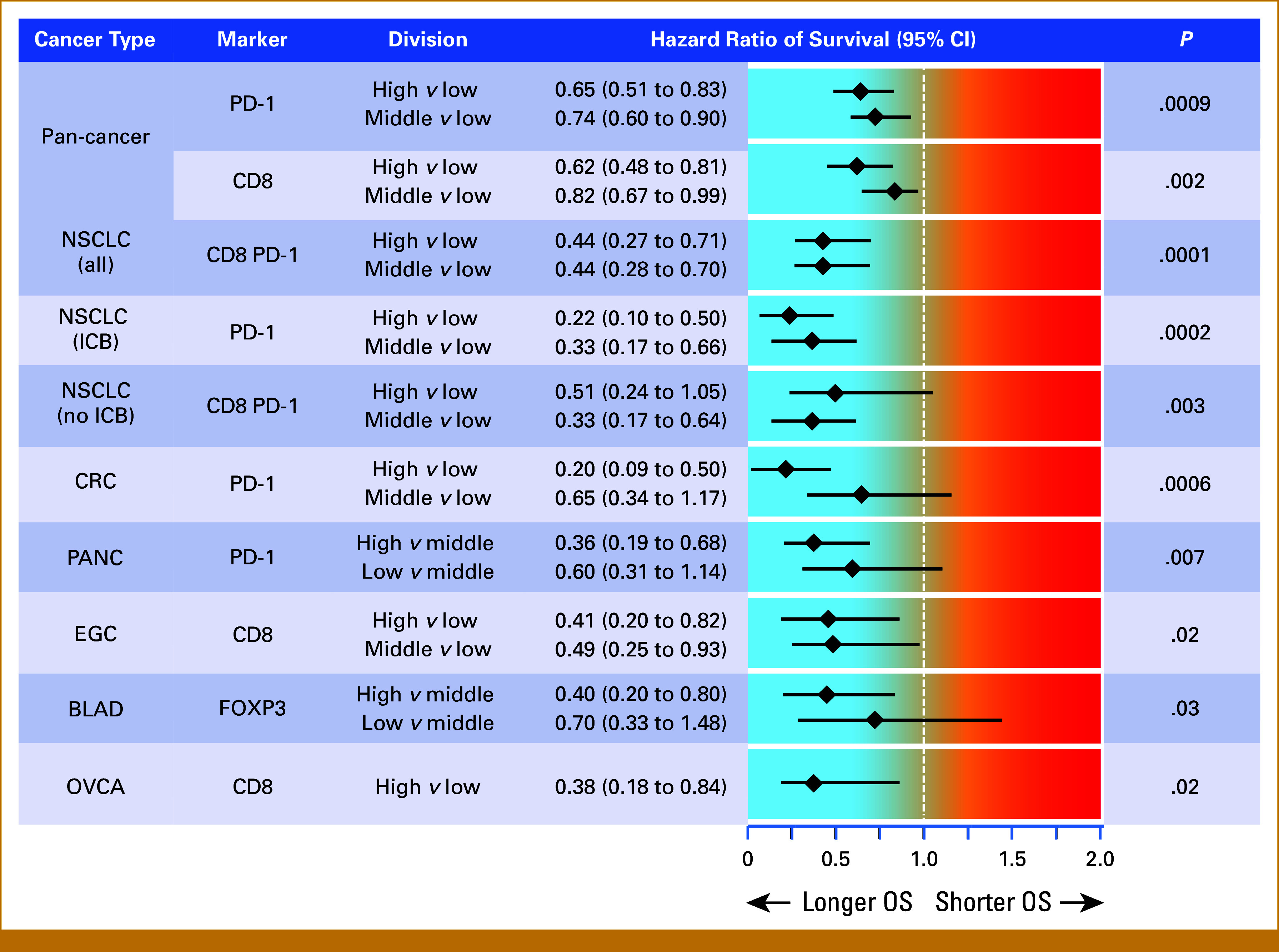
Risk of death in multivariable biomarker Cox models with clinical risk factor adjustments for cancers with high case numbers. The clinical risk factors considered in the multivariable modeling for each group are listed in Table 1. BLAD, bladder carcinoma; CRC, colorectal carcinoma; cut. MEL, cutaneous melanoma; EGC, esophagogastric carcinoma; ICB, treatment with immune checkpoint blockade; no-ICB, no treatment with immune checkpoint blockade; NSCLC, non–small cell lung cancer; OS, overall survival; OVCA, ovarian carcinoma; PANC, pancreatic carcinoma.

For the pan-cancer cohort, patients with high PD-1^+^ or high CD8^+^ cell counts had lower risks of death, which were independently prognostic (Fig 4). There was 35% lower risk of death for those with high numbers of PD-1^+^ cells (*P* = .0009) and a similar result for CD8^+^ cells (*P* = .002, Fig 4).

In cancer type–specific analyses, only a single biomarker was the best, independent predictor of the reduced risk of death. Patients with NSCLC with high intratumoral CD8^+^PD-1^+^ cells had a 56% reduced risk of death independent of clinical risk factors (*P* = .0001, Fig 4). Patients with CRC with high intratumoral PD-1^+^ cells had 80% reduced risk of death independent of additional risk factors (*P* = .0006, Fig 4). Patients with EGC with high CD8^+^ cells had a 59% reduction in risk (*P* = .02). Patients with OVCA with high CD8^+^ cells had a 62% reduction in risk (*P* = .02, Fig 4). Patients with PANC and high PD-1^+ ^counts and patients with BLAD and high FOXP3^+^ cell counts had, respectively, 64% and 68% reduced risk compared with those with intermediate counts (*P* = .03, Fig 4, Data Supplement, Table S5).

### Univariable Analyses Show That Select Immune Cell Populations Are Most Strongly Linked to OS

**FIG 5.**
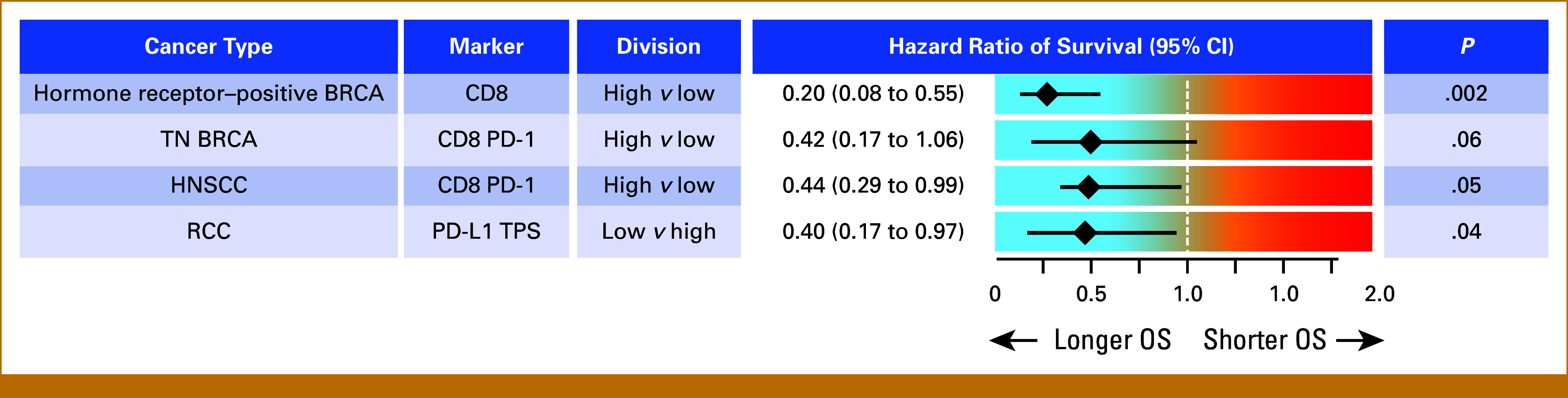
Risk of death in univariable biomarker Cox models with clinical risk factor adjustments for cancers with low case numbers. The clinical risk factors considered in the univariate modeling for each group are listed in Table 1. HNSCC, head and neck squamous cell carcinoma; OS, overall survival; RCC, renal cell carcinoma; TN BRCA, triple-negative breast carcinoma; TPS, tumor proportion score.

We did not attempt multivariable biomarker analysis for patients with cancer types that resulted in fewer than 30 deaths. Instead, we calculated the risk of death using the biomarker most significantly associated with OS after adjusting for clinical risk factors (as shown in Table 1). Patients with hormone receptor–positive/HER2– BRCA with high CD8^+^ cell counts had an 80% lower risk of death compared with those with low counts (*P* = .002, Fig 5). Patients with TN BRCA with high CD8^+^PD-1^+^ cell counts had a lower risk that approached significance (*P* = .06; Fig 5). Patients with HNSCC with high CD8^+^PD-1^+^ cell counts had a 56% lower risk (*P* = .05). Finally, patients with RCC with *low* TPS had a 60% lower risk compared with those with *high* TPS (*P* = .04, Fig 5).

## DISCUSSION

Expert visual interpretation of cancer tissue histomorphology on H&E-stained sections remains the cornerstone of cancer diagnosis.^1,2^ The results are often supplemented with selective IHC staining to establish tumor cell lineage and targeted genetic testing to detect specific oncogenic drivers.^4,42^ However, tissue sections contain additional biological information that is not routinely captured or reported.

We have addressed these deficiencies by validating and prospectively testing ImmunoProfile, a new multistep workflow that incorporates automated mIF staining, WSI, and ML-assisted cell quantitation to reliably capture and quantify specific immune biomarkers in FFPE tissue samples in the routine clinical laboratory. The results were concordant with traditional IHC, reproducible over time, and yielded reportable data in 97% of the >2,000 cases tested prospectively over 3.25 years. Thus, we find ImmunoProfile to be as robust and reliable as the traditional IHC assays currently used in clinical laboratories.

This study was not constructed or intended as a trial to find optimal biomarker thresholds. Rather, ImmunoProfile was conceived and executed to test the feasibility and significance of prospectively implementing a clinically validated biomarker test in all cases that undergo pathologic review at a tertiary cancer center. Remarkably, we consistently found robust associations between high numbers of tumor-infiltrating CD8^+^, PD-1^+^, and/or CD8^+^PD-1^+^ immune cells and superior patient survival across cancer types, despite the heterogeneity in tissues, cancers, patient stage, and therapies. Moreover, they remained highly significant in predicting patient survival in Cox models accounting for major clinical risk factors.

The survival advantage for patients with high numbers of intratumoral PD-1^+^ cells might be unexpected. However, PD-1, a marker of both T-cell activation and exhaustion, may serve as a more reliable marker of ongoing antitumor immunity than CD8 alone.^43^ Moreover, recent publications suggest that PD-1^+^ and CD8^+^PD-1^+^ immune cells may represent tumor antigen–specific T cells.^44-46^

Unexpectedly, we found that patients with BLAD and high numbers of FOXP3^+^ cells had longer OS. We also found that patients with RCC and *low* PD-L1 TPS scores (below the group median) had longer OS. As these counterintuitive results have been found in previous studies, they likely reflect a unique immunobiology inherent to these tumors.^41,47^

Our study, data, and analyses have several limitations, as expected for prospective data collection on an unselected patient population. Thus, the precise survival benefit for an individual patient treated in a specific manner may not be reliably determined from these current data. Acquiring such specific information will require the broad implementation of the quantitative assay in clinical laboratories and its application to rigorously defined, treated, and followed patient populations. Our decisions regarding which and how many risk factors to include were informed choices based on cancer cohort sizes, the relative clinical importance of individual risk factors, and our available clinical information. Since readers may prioritize other parameters and wish to create their own survival curves, we have provided the raw data in the Data Supplement (Table S1). For future reference and subsequent validation studies, we also provide the optimal thresholds and their corresponding hazard ratios (HRs) for each significant immune biomarker in the Data Supplement (Table S6).

In summary, the real-time quantitative evaluation of defined intratumoral immune cell populations using a clinically validated assay, particularly the enumeration of CD8^+^, PD-1^+^, and CD8^+^PD-1^+^ cells, provides prognostic information across various cancer types, independent of the current staging and risk assessment systems. In this way, ImmunoProfile both validates and extends the validation of Immunoscore, a semi-automated staining, imaging, and scoring platform that has undergone prospective testing and has been shown to improve the stratification of patients with colon carcinoma more effectively than the AJCC TNM staging system alone.^22,24^ With the advances in automated staining, imaging, and scoring technologies, we can anticipate that these and additional, highly specified lymphoid subsets identified in research studies^48^ will be reliably captured and reported in routine clinical practice soon.

## Data Availability

A data sharing statement provided by the authors is available with this article at DOI https://doi.org/10.1200/PO-25-00240.
